# Clinical Outcomes and Quality of Life in Recipients of Livers Donated after Cardiac Death

**DOI:** 10.1155/2015/680316

**Published:** 2015-04-01

**Authors:** Neehar D. Parikh, Anton I. Skaro, Daniela P. Ladner, Vadim Lyuksemburg, Joshua G. Cahan, Amna Daud, Zeeshan Butt

**Affiliations:** ^1^Division of Gastroenterology, University of Michigan, Ann Arbor, MI 48109, USA; ^2^Division of Gastroenterology and Hepatology, Northwestern University, Chicago, IL 60611, USA; ^3^Northwestern University Transplant Outcomes Research Collaborative (NUTORC), Northwestern University, Chicago, IL 60611, USA; ^4^Comprehensive Transplant Center, Feinberg School of Medicine, Northwestern University, Chicago, IL 60611, USA; ^5^Department of Medical Social Sciences, Northwestern University, Chicago, IL 60611, USA

## Abstract

Donation after cardiac death (DCD) has expanded in the last decade in the US; however, DCD liver utilization has flattened in recent years due to poor outcomes. We examined clinical and quality of life (QOL) outcomes of DCD recipients by conducting a retrospective and cross-sectional review of patients from 2003 to 2010. We compared clinical outcomes of DCD recipients (*n* = 60) to those of donation after brain death (DBD) liver recipients (*n* = 669) during the same time period. DCD recipients had significantly lower rates of 5-year graft survival (*P* < 0.001) and a trend toward lower rates of 5-year patient survival (*P* = 0.064) when compared to the DBD cohort. In order to examine QOL outcomes in our cohorts, we administered the Short Form Liver Disease Quality of Life questionnaire to 30 DCD and 60 DBD recipients. The DCD recipients reported lower generic and liver-specific QOL. We further stratified the DCD cohort by the presence of ischemic cholangiopathy (IC). Patients with IC reported lower QOL when compared to DBD recipients and those DCD recipients without IC (*P* < 0.05). While the results are consistent with clinical experience, this is the first report of QOL in DCD recipients using standardized measures. These data can be used to guide future comparative effectiveness studies.

## 1. Introduction

Over the past decades, outcomes after liver transplantation (LT) have continuously improved with more than 80% one-year patient survival along with significant improvements in recipient quality of life (QOL) [1, 2]. Unfortunately, with the severe organ shortage in the United States, over 16,000 patients are currently waiting for a LT [2]. Consequently, depending on the donation service area, approximately 10–30% of LT candidates are removed from the waitlist prior to receiving a LT, due to either death or deteriorating medical condition [3].

In 2003, the Health Resources and Services Administration (HRSA), the Centers for Medicare and Medicaid Services (CMS), and several organ procurement organizations formed a collaboration to formulate strategies to expand the donor pool. One strategy offered by the group was to increase the use of organs donated after cardiac death (DCD), which consequently led to an expansion in the number of DCD LTs in the US [4]. However, increased use of DCD grafts has well-documented negative medical sequelae, which have resulted in flattening in the growth of DCD liver utilization in the US [5].

Patients who receive a DCD LT are at increased risk for ischemic cholangiopathy (IC), which typically manifests within one year of transplantation [6]. Those patients with IC may experience significant symptoms such as itching, jaundice, recurrent infections, and severe fatigue [7, 8]. Furthermore, IC leads to higher rates of graft failure and patient death [7, 9, 10]. To help relieve these symptoms, recipients often require repeated percutaneous or endoscopic invasive biliary procedures to relieve biliary obstructions [7]. Once diffuse IC is identified, there is typically no effective cure except for a repeat LT, which is often difficult due to a lack of priority within the current model of end stage liver disease- (MELD-) based liver allocation system [7]. The prevalence of complications, the need for recurrent procedures, and possible requirement of a repeat LT in patients with IC prevent the patient from returning to a normal life after LT and may have profound psychosocial consequences [11]. While the medical sequelae of DCD LT have been explored and established in the published literature [7, 12], the impact of the receipt of a DCD LT on recipient QOL has not yet been described.

In its broadest sense, QOL can be defined as “an overall sense of well-being, including aspects of happiness and satisfaction with life as a whole” [13]. This definition, proposed by the Centers for Disease Control and Prevention, includes specific, measurable concepts such as mental well-being, physical functioning, and overall health status, which may be influenced by multiple factors, such as occupational and marital status [13]. Ideally, quality of life data serve as a complementary endpoint in transplantation research that can help contextualize medical outcomes such as survival and medical morbidity.

The aims of this study were to describe medical and QOL outcomes of DCD recipients and compare them with outcomes of donation after brain death (DBD) recipients from a single center cohort. We hypothesized that clinical outcomes and patient-reported QOL would be worse in our DCD cohort. We further hypothesized that those who experience IC after LT would experience the worst QOL.

## 2. Methods

### 2.1. Medical Outcomes

We conducted a retrospective analysis of our institutional LT outcomes, stratified by DCD and DBD organs from January 1, 2003, and June 1, 2010. Recipient demographic, laboratory, and outcomes data were obtained from the electronic medical record for both DCD and DBD cohorts. We excluded patients who underwent live-donor liver transplantation, concomitant pancreatic or small bowel transplantation, or repeat transplantation and in whom the index LT was performed prior to January 1, 2003. The primary medical outcome of interest was graft failure, which was defined as patient death or retransplantation. Secondary outcomes included graft loss, patient death, time to graft loss, and time to death in the DCD and DBD cohorts. The same procurement method was applied for all DCD LT [14]. All DCD organ recoveries occurred in a “controlled” manner (Maastricht Category 3). Support was withdrawn in either the ICU or operating room. Declaration of death, according to cessation of cardiopulmonary activity, was followed by a mandatory 5-minute waiting period. WIT was measured from drop in systolic blood pressure to <50 mm Hg or oxygen saturation <70% to cannulation and cold perfusion [14]. Our center considered all available DCD donors at the time organ offer and if they fulfilled Maastricht Category 3 criteria, the liver allografts were considered for transplantation.

### 2.2. QOL Assessment

A trained research assistant contacted all surviving DCD recipients prior to their next transplant clinic appointment. After providing informed consent, eligible patients completed the QOL survey. Eligible patients who did not have a scheduled clinic appointment were contacted by phone. In those cases, if the patient verbally agreed, the consent and survey were reviewed over the phone by trained research staff, as approved by our Institutional Review Board (IRB). For DBD recipients, we used a convenience sample of patients consenting and surveyed prior to a postoperative transplant clinic appointment. We individually matched the DBD recipients to DCD recipients (2 : 1 ratio) using the following variables: age, sex, race, pre-LT MELD, cause of liver disease, and time since LT (±30 days). Patient demographic, laboratory, and patient procedure data were obtained from the electronic health records with consent of the patient and approval from our IRB.

### 2.3. QOL Measure

Liver recipients completed the Short Form Liver Disease Quality of Life (SF-LDQOL), a disease-targeted QOL instrument designed for patients with chronic liver disease [15]. The SF-LDQOL contains subscales specific to liver disease, including chronic liver disease related symptoms, chronic liver disease related effects on activities of daily living, concentration, memory, quality of social interaction, health distress, sleep, hopelessness, loneliness, self-perceived stigma of chronic liver disease, sexual functioning, and sexual problems. Scores were created by averaging the items on these subscales and subsequent linear transformation to a possible score of 0–100 [15]. The SF-LDQOL also contains the Short Form-36 (SF-36), a widely used generic QOL instrument that contains health subscales assessing physical functioning (PF), role physical (RP; limitations due to physical health), bodily pain (BP), general health (GH), vitality (VT), social functioning (SF), role emotional (RE; limitations related to emotional problems), and mental health (MH). The subscales can also be summarized into a physical and mental component summary score. Three scales (PF, RP, and BP) contribute most to the scoring of the physical component score (PCS). The MH, RE, and SF scales contribute most to the scoring of the mental component score (MCS). The remaining scales have contributions to both summary measures. General population norm based scores are standardized to a mean of 50 and SD of 10 [15].

### 2.4. Statistical Analysis

We used the independent sample *t*-test and Pearson's chi-square test to assess differences in baseline characteristics and QOL scores of the DCD and DBD cohorts. The variables assessed included age, race, etiology of liver disease, MELD at LT, donor characteristics, and time since LT. We performed further comparisons for significant baseline differences between the groups and the QOL scores. In order to compare the time course of patient outcomes in DCD and DBD cohorts, we constructed a Kaplan-Meier curve with time to graft loss and time to patient death for each cohort with the Mantel-Cox log rank test to determine significance.

Within the DCD cohort, we further stratified based upon the presence of IC. The independent sample *t*-test and Pearson's chi-square test were used to compare the baseline differences and QOL scores in these populations as well. We also conducted an ANOVA to determine significance between group differences between the DBD, DCD without IC, and DCD with IC cohorts.

We constructed a stepwise logistic regression using age, MELD at LT, hepatitis C (HCV) cirrhosis as a cause of the liver disease, and presence of a combined liver-kidney transplant to determine whether the covariates accounted for differences in recipient QOL scores.

## 3. Results

### 3.1. Medical Outcomes

Between January 1, 2003, and June 1, 2010, there were a total of 60 DCD LTs and 669 DBD LTs performed at our center. The mean age of the entire sample was 54.7 (±10.4), and the sample was mostly male (63.8%) and Caucasian (75.8%). The most common blood types were A and O (38.7% each) and the average calculated MELD at the time of LT was 24.9 (±11.0). The baseline characteristics of the recipient and donor population stratified by donor type are shown in Table 1. Of note, the DBD population had a significantly higher MELD score and rate of simultaneous liver kidney transplantation.

**Figure 1:**
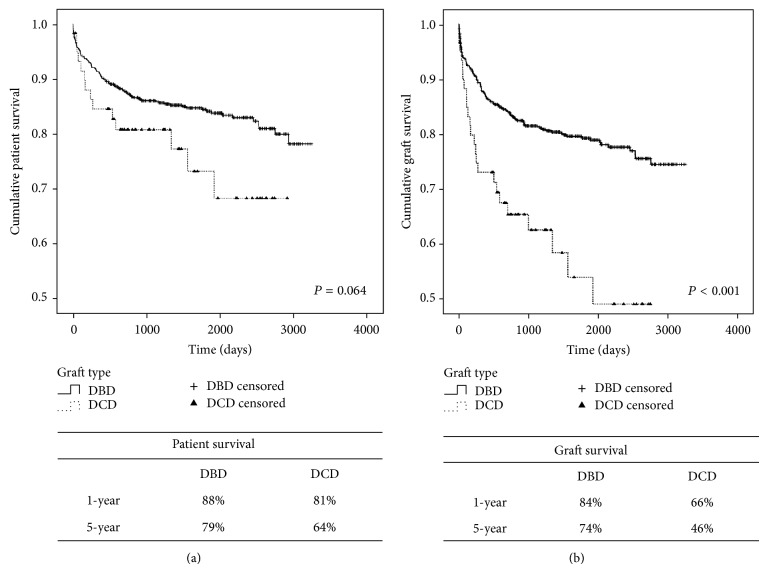
Kaplan-Meier curves of (a) time to death and (b) time to graft failure. DCD: donation after cardiac death and DBD: donation after brain death.

**Figure 2:**
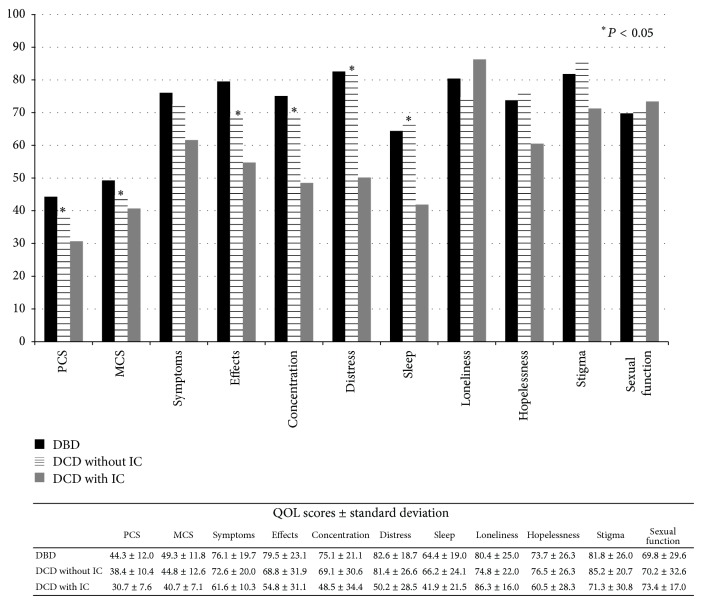
QOL scores in cohorts from the Short Form Liver Disease Quality of Life instrument. PCS: physical composite score, MCS: mental composite score, DCD: donation after cardiac death, DBD: donation after brain death, and IC: ischemic cholangiopathy.

The DCD population had significantly higher rates of ischemic cholangiopathy (*P* < 0.001), graft loss (*P* < 0.001), and patient death (*P* < 0.001) when compared to the DBD population. The time to event analysis for graft loss and patient death showed a significantly more rapid rate of graft failure for the DCD population (*P* < 0.001) and a trend towards more rapid patient death (*P* = 0.064) (Figure 1). The mean time to graft loss in the DCD population was 417 (±108) days versus 565 (±57) days for the DBD population (*P* = NS). The mean time to death was 450 (±54) days for the DCD cohort versus 580 (±176) days for the DBD cohort (*P* = NS). The 1-year and 5-year patient survival was 88% and 79% for the DBD cohort and 81% and 64% for the DCD cohort (*P* = NS) (Figure 2). The 1-year and 5-year graft survival was 84% and 74% for the DBD cohort and 66% and 46% for the DCD cohort (*P* < 0.001) (Figure 2).

### 3.2. Quality of Life Assessment

A total of 36 DCD recipients were living and had not been retransplanted at the time of the survey administration, and 30 (83%) consented to completing a QOL assessment. There were no significant differences in baseline characteristics (age, MELD at LT, etiology of liver disease, and sex) of the patients who participated in the QOL survey to those who refused (*P* = NS). In an effort to accurately compare DCD and DBD recipients, we constructed a matched sample, based on age, sex, race, pre-LT MELD, cause of liver disease, and time since LT, of 60 patients who received DBD livers. The baseline characteristics of the two groups are shown in Table 2. There were no significant differences in age, race distribution, rate of HCV cirrhosis, calculated MELD score at the time of LT, or time since LT. Additionally there was no significant difference in blood type distribution between the DCD and DBD cohorts. The proportion of patients who received a combined liver-kidney transplant was significantly lower in the DCD cohort (*P* < 0.05).

The QOL summary and subscale scores in the cohorts are shown in Figure 2. In the DCD cohort as whole, the physical and mental QOL scores were significantly lower (*P* < 0.05) when compared to the DBD cohort. The liver-specific subscale scores, which measure the chronic liver disease related effects on activities of daily living and concentration, were also lower in the DCD cohort (*P* < 0.05). After adjustment for age, MELD at LT, and presence of HCV induced liver disease and presence of a liver-kidney transplant, the regression analysis showed significant differences in QOL scores between the DCD and DBD cohorts.

Given the higher rates of biliary complications seen in patients with ischemic cholangiopathy, we performed an exploratory analysis to examine the QOL scores within the DCD cohort after stratifying by the presence of IC (*n* = 8) (Figure 2). There were no significant differences in baseline characteristic or time since LT between those DCD recipients with or without IC. Our DBD sample with QOL data did not experience IC. On ANOVA, there were significant QOL differences between the DBD, DCD without IC, and the DCD with IC groups. Specifically, differences emerged on the MCS, PCS, chronic liver disease related effects on activities of daily living, concentration, health distress, and sleep domains (Figure 2). In the independent samples *t*-test analysis, those patients who developed post-LT IC had lower QOL scores (*P* < 0.05) when compared to DCD without IC, driving the lower QOL score seen in the entire DCD cohort. The IC cohort also had significantly lower scores in the health distress and sleep subscales of the SF-LDQOL when compared to the DCD without IC cohort (*P* < 0.05). There were no significant differences in QOL scores between the DCD cohort without IC and the DBD cohort (*P* = NS).

## 4. Discussion

The use of DCD liver for transplantation has dramatically increased since 2003, when HRSA/CMS endorsed DCD LT to increase available organs. Our institutional patient and graft survival outcomes overall showed inferior graft and patient survival in the DCD cohort when compared to the DBD cohort. The time to graft failure was also significantly more rapid in the DCD cohort. Our institutional results with DCD LT are worse than those previously reported at other centers [6, 12]. In the largest institutional data reported to date (*n* = 200), Taner et al. found that 5-year graft survival in their DCD LT cohort was 69%, which is higher than our institutional 5-year graft survival (46%) [16]. Notably, our 5-year DCD patient survival rate was also markedly less at 64% [16]. However, our outcomes are comparable to other reported centers described in a published meta-analysis and review of the national registry [9, 10].

To our knowledge, we are the first center to report QOL for DCD compared to DBD recipients or the impact of IC on QOL. Specifically, we explored QOL and clinical outcomes in patients who received DCD organs and compared them to a cohort of DBD recipients at our institution. Our DCD recipients showed significantly worse physical and emotional well-being, as indexed by PCS and MCS scores, when compared to a subset of our DBD recipients. Given the typical time course of DCD graft failures (i.e., usually within 1 year of LT), these data may actually underestimate the poorer QOL experienced by DCD liver recipients.

Interestingly, those DCD patients with IC drove the lower QOL scores seen in our cohort; DCD patients who did not experience IC were comparable to our DBD cohort on these measures. While it is true that these results are consistent with clinical wisdom, our data is the first to demonstrate the specific QOL domains and the measured magnitude of differences seen between these groups. Such data may be useful in planning future comparative effectiveness trials involving DCD and DBD cohorts or for studies that aim to reduce the incidence of IC, for example. Without actual data from standardized health status tools, we miss opportunities for improving scientific rigor where such information directly relates to patient's life quality.

Volk and Hagan published a study investigating the correlation between recipient QOL and organ quality as measured by donor risk index (DRI). The authors found no significant correlation between DRI and scores on the SF-36, a generic QOL measure [11]. Notably, DCD status is a component of the DRI; however, given that DRI is a composite of several factors, it is unclear how many DCD recipients the Volk and Hagan cohort contained and to what extent DCD status affected patient QOL [17]. Other studies have shown that DCD liver transplant is associated with more intensive resource utilization (procedures, office visits) which may directly affect QOL [14].

The liver-specific QOL measures are difficult to interpret within our study, given that the scale was developed in a chronic liver disease population rather than a post-LT population [15]. Theoretically, though, the liver-specific scales of the SF-LDQOL might be more sensitive to issues following LT. This assumption requires further validation in this population. In addition, no normative values for the subscales have been established, making definitions of adequate or inadequate symptom control difficult. Nonetheless, in our sample, we found significant differences in select liver-specific subscales in both the DCD populations, with the most pronounced differences observed in the subset of patients with IC.

Our study has a number of limitations. Although our DCD LT center outcomes are consistent with published reports from other centers [10], our outcomes are worse than those reported in the largest published series [16]. This is in part due to our institutional practice of aggressively retransplanted patients with severe IC and reflects regional differences in organ availability for retransplantation. Our cross-sectional design only allowed for measurement of recipient QOL at one time point. Baseline pre-LT QOL measures were not available for both the DBD and DCD cohorts. A longitudinal study may better capture the natural course of QOL experienced in this population. In addition, we studied relatively few DCD recipients. Although we did show significant QOL differences between DCD and DBD recipients, sample sizes may have limited our ability to detect other significant differences. In addition, we were only able to capture QOL data on all of our DCD recipients. While there were no clinical differences seen between those patients who provided QOL data and those who did not, our results may have been affected by sampling bias. Consequently, these findings should be considered exploratory in nature and require further confirmation in a larger cohort of patients from other institutions. Finally, the SF-LDQOL was not designed to evaluate QOL in a post-LT population. Future QOL studies in LT should consider utilizing a measure specifically designed to assess post-LT recovery, such as the QOL instrument published by Saab et al., which was not available at the time our study was initiated [18]. The primary strength of this study was the inclusion of a QOL component to the overall outcomes of our DCD cohort.

In conclusion, we found inferior quality of life, graft survival, and patient survival in our patients who received DCD liver transplants. Strikingly, only a minority of patients (36.7%) had an overall adequate outcome (based upon graft survival and QOL measurement). These findings need to be tempered against the substantial competing risk of waitlist mortality. In addition, the inferior outcomes that are seen with DCD must be discussed with patients during the informed consent process. This is a difficult task given the complex decision making that goes into organ acceptance; however, as Volk et al. have recently shown, there are innovative ways to involve patients in the shared decision making regarding organ quality [19]. There are evolving technologies to improve outcomes in DCD donors by reducing the impact of warm ischemia time. Several groups have published improvement in outcomes of DCD liver donors with the use of ex vivo machine perfusion in animal models [20–22]. The application of these technologies to human DCD organ recovery could decrease the incidence of ischemic cholangiopathy and improve quality of life after LT.

Moving forward, prospective studies should be conducted to confirm our results and better understand the time course of recipient QOL depending on organ quality. In addition, future trials should focus on interventions, such as psychosocial support or physical therapy, to alleviate the poor QOL seen in this subset of the recipient population.

## Figures and Tables

**Table 1:** Baseline characteristics of liver transplant recipients.

	DCD (*n* = 60)	DBD (*n* = 669)	*P* value
Age	55.2 ± 11.8	54.6 ± 10.3	*P* = 0.38
Gender (% male)	71.7%	63.1%	*P* = 0.19
Calculated MELD at LT	21.1 ± 9.9	25.3 ± 11.0	*P* = 0.03
MELD upgrade at LT (%)	35.6%	40.0%	*P* = 0.49
Etiology of liver disease			
HCV	26.7%	25.5%	*P* = 0.66
EtOH	20%	18.7%	
HCV/EtOH	15%	11.7%	
NASH/cryptogenic	15.0%	16.6%	
Fulminant	6.7%	4.2%	
HCC at LT	36.7%	29.1%	*P* = 0.21
Liver-kidney transplant	5.0%	21.2%	*P* = 0.003
Cold ischemia time (hrs)	5.6 ± 1.6	5.4 ± 1.8	*P* = 0.68
Warm ischemia time (min)	16.3 ± 5.4		
Ischemic cholangiopathy	23%	0.2%	*P* < 0.001
Graft failure	40.0%	19.6%	*P* < 0.001
Death	28.3%	16.4%	*P* < 0.001

DCD: donation after cardiac death, DBD: donation after brain death, MELD: model of end stage liver disease, LT: liver transplantation, HCV: hepatitis C Virus, EtOH: ethanol, NASH: nonalcoholic steatohepatitis, and HCC: hepatocellular carcinoma.

**Table 2:** Baseline characteristics of liver transplant recipients with a quality of life assessment.

	DCD (*n* = 30)	DBD (*n* = 60)	
Age at survey	54.1 ± 14.1	51.7 ± 13.4	*P* = 0.32
Gender (% male)	67.6%	65.4%	*P* = 0.34
Calculated MELD at liver transplantation	22.4 ± 10.2	20.7 ± 10.7	*P* = 0.11
MELD upgrade at liver transplantation (%)	38.6%	34.2%	*P* = 0.38
Etiology of liver disease			
HCV	30.3%	32.1%	*P* = 0.64
EtOH	16%	12.7%	
HCV/EtOH	6.9%	9.9%	
NASH/cryptogenic	12.8%	15.8%	
Fulminant	4.2%	3.1%	
HCC at liver transplantation	29.3%	26.4%	*P* = 0.37
Liver-kidney transplant	3%	17.9%	*P* < 0.001
Cold ischemia time (hrs)	5.8 ± 1.4	5.3 ± 1.7	*P* = 0.24
Warm ischemia time (min)	15.1 ± 5.8		
Time since liver transplantation (days)	654 ± 219	1014 ± 608	*P* = 0.071
Ischemic cholangiopathy	26.6%	0%	*P* < 0.001

DCD: donation after cardiac death, DBD: donation after brain death, MELD: model of end stage liver disease, LT: liver transplantation, HCV: hepatitis C virus, EtOH: ethanol, NASH: nonalcoholic steatohepatitis, and HCC: hepatocellular carcinoma.

## Abbreviations

BP: Bodily pain
CMS: Centers for Medicare and Medicaid Services
DBD: Donation after brain death
DCD: Donation after cardiac death
EtOH: Ethanol
GH: General health
HRSA: Health Resources and Services Administration
HCV: Hepatitis C virus
HCC: Hepatocellular carcinoma
IC: Ischemic cholangiopathy
LT: Liver transplantation
MCS: Mental component score
MH: Mental health
MELD: Model of end stage liver disease
NASH: Nonalcoholic steatohepatitis
PCS: Physical component score
PF: Physical functioning
QOL: Quality of life
RE: Role emotional
RP: Role physical
SF-36: Short Form-36
SF-LDQOL: Short Form Liver Disease Quality of Life
SF: Social functioning
VT: Vitality.

## References

[1] Tome S., Wells J. T., Said A., Lucey M. R. (2008). Quality of life after liver transplantation. A systematic review. *Journal of Hepatology*.

[3] Washburn K., Pomfret E., Roberts J. (2011). Liver allocation and distribution: possible next steps. *Liver Transplantation*.

[4] Shafer T. J., Wagner D., Chessare J. (2008). US organ donation breakthrough collaborative increases organ donation. *Critical Care Nursing Quarterly*.

[5] Orman E. S., Barritt A. S., Wheeler S. B., Hayashi P. H. (2013). Declining liver utilization for transplantation in the United States and the impact of donation after cardiac death. *Liver Transplantation*.

[6] Foley D. P., Fernandez L. A., Leverson G. (2011). Biliary complications after liver transplantation from donation after cardiac death donors: an analysis of risk factors and long-term outcomes from a single center. *Annals of Surgery*.

[7] Skaro A. I., Jay C. L., Baker T. B. (2009). The impact of ischemic cholangiopathy in liver transplantation using donors after cardiac death: the untold story. *Surgery*.

[8] Detry O., Donckier V., Lucidi V. (2010). Liver transplantation from donation after cardiac death donors: initial Belgian experience 2003–2007. *Transplant International*.

[9] Jay C., Ladner D., Wang E. (2011). A comprehensive risk assessment of mortality following donation after cardiac death liver transplant—an analysis of the national registry. *Journal of Hepatology*.

[10] Jay C. L., Lyuksemburg V., Ladner D. P. (2011). Ischemic cholangiopathy after controlled donation after cardiac death liver transplantation: a meta-analysis. *Annals of Surgery*.

[11] Volk M. L., Hagan M. (2011). Organ quality and quality of life after liver transplantation. *Liver Transplantation*.

[12] de Vera M. E., Lopez-Solis R., Dvorchik I. (2009). Liver transplantation using donation after cardiac death donors: long-term follow-up from a single center. *American Journal of Transplantation*.

[13] Centers for Disease Control and Prevention (2000). *Measuring Healthy Days*.

[14] Jay C. L., Lyuksemburg V., Kang R. (2010). The increased costs of donation after cardiac death liver transplantation: caveat emptor. *Annals of Surgery*.

[15] Kanwal F., Spiegel B. M. R., Hays R. D. (2008). Prospective validation of the short form liver disease quality of life instrument. *Alimentary Pharmacology and Therapeutics*.

[16] Taner C. B., Bulatao I. G., Willingham D. L. (2012). Events in procurement as risk factors for ischemic cholangiopathy in liver transplantation using donation after cardiac death donors. *Liver Transplantation*.

[17] Feng S., Goodrich N. P., Bragg-Gresham J. L. (2006). Characteristics associated with liver graft failure: the concept of a donor risk index. *The American Journal of Transplantation*.

[18] Saab S., Ng V., Landaverde C. (2011). Development of a disease-specific questionnaire to measure health-related quality of life in liver transplant recipients. *Liver Transplantation*.

[19] Volk M. L., Tocco R. S., Pelletier S. J., Zikmund-Fisher B. J., Lok A. S. F. (2011). Patient decision making about organ quality in liver transplantation. *Liver Transplantation*.

[20] Fondevila C., Hessheimer A. J., Maathuis M.-H. J. (2011). Superior preservation of DCD livers with continuous normothermic perfusion. *Annals of Surgery*.

[21] Xu H., Berendsen T., Kim K. (2012). Excorporeal normothermic machine perfusion resuscitates pig DCD livers with extended warm ischemia. *Journal of Surgical Research*.

[22] Boehnert M. U., Yeung J. C., Bazerbachi F. (2013). Normothermic acellular ex vivo liver perfusion reduces liver and bile duct injury of pig livers retrieved after cardiac death. *American Journal of Transplantation*.

